# Risk Assessment Models for Venous Thromboembolism in Medical Inpatients

**DOI:** 10.1001/jamanetworkopen.2024.9980

**Published:** 2024-05-10

**Authors:** Emmanuel Häfliger, Basil Kopp, Pauline Darbellay Farhoumand, Damien Choffat, Jean-Benoît Rossel, Jean-Luc Reny, Drahomir Aujesky, Marie Méan, Christine Baumgartner

**Affiliations:** 1 Department of General Internal Medicine, Inselspital, Bern University Hospital, University of Bern, Bern, Switzerland; 2 Division of General Internal Medicine, Department of Medicine, Geneva University Hospitals, Geneva, Switzerland; 3 Division of Internal Medicine, Department of Medicine, Lausanne University Hospital, Lausanne, Switzerland; 4 CTU Bern, University of Bern, Bern, Switzerland

## Abstract

**Importance:**

Thromboprophylaxis is recommended for medical inpatients at risk of venous thromboembolism (VTE). Risk assessment models (RAMs) have been developed to stratify VTE risk, but a prospective head-to-head comparison of validated RAMs is lacking.

**Objectives:**

To prospectively validate an easy-to-use RAM, the simplified Geneva score, and compare its prognostic performance with previously validated RAMs.

**Design, Setting, and Participants:**

This prospective cohort study was conducted from June 18, 2020, to January 4, 2022, with a 90-day follow-up. A total of 4205 consecutive adults admitted to the general internal medicine departments of 3 Swiss university hospitals for hospitalization for more than 24 hours due to acute illness were screened for eligibility; 1352 without therapeutic anticoagulation were included.

**Exposures:**

At admission, items of 4 RAMs (ie, the simplified and original Geneva score, the Padua score, and the IMPROVE [International Medical Prevention Registry on Venous Thromboembolism] score) were collected. Patients were stratified into high and low VTE risk groups according to each RAM.

**Main Outcomes and Measures:**

Symptomatic VTE within 90 days.

**Results:**

Of 1352 medical inpatients (median age, 67 years [IQR, 54-77 years]; 762 men [55.4%]), 28 (2.1%) experienced VTE. Based on the simplified Geneva score, 854 patients (63.2%) were classified as high risk, with a 90-day VTE risk of 2.6% (n = 22; 95% CI, 1.7%-3.9%), and 498 patients (36.8%) were classified as low risk, with a 90-day VTE risk of 1.2% (n = 6; 95% CI, 0.6%-2.6%). Sensitivity of the simplified Geneva score was 78.6% (95% CI, 60.5%-89.8%) and specificity was 37.2% (95% CI, 34.6%-39.8%); the positive likelihood ratio of the simplified Geneva score was 1.25 (95% CI, 1.03-1.52) and the negative likelihood ratio was 0.58 (95% CI, 0.28-1.18). In head-to-head comparisons, sensitivity was highest for the original Geneva score (82.1%; 95% CI, 64.4%-92.1%), while specificity was highest for the IMPROVE score (70.4%; 95% CI, 67.9%-72.8%). After adjusting the VTE risk for thromboprophylaxis use and site, there was no significant difference between the high-risk and low-risk groups based on the simplified Geneva score (subhazard ratio, 2.04 [95% CI, 0.83-5.05]; *P* = .12) and other RAMs. Discriminative performance was poor for all RAMs, with an area under the receiver operating characteristic curve ranging from 53.8% (95% CI, 51.1%-56.5%) for the original Geneva score to 58.1% (95% CI, 55.4%-60.7%) for the simplified Geneva score.

**Conclusions and Relevance:**

This head-to-head comparison of validated RAMs found suboptimal accuracy and prognostic performance of the simplified Geneva score and other RAMs to predict hospital-acquired VTE in medical inpatients. Clinical usefulness of existing RAMs is questionable, highlighting the need for more accurate VTE prediction strategies.

## Key Points

**Question:**

What is the prognostic performance of the simplified Geneva score and other validated risk assessment models (RAMs) to predict venous thromboembolism (VTE) in medical inpatients?

**Findings:**

In this cohort study providing a head-to-head comparison of validated RAMs among 1352 medical inpatients, sensitivity of RAMs to predict 90-day VTE ranged from 39.3% to 82.1% and specificity of RAMs ranged from 34.3% to 70.4%. Discrimination was poor, with an area under the receiver operating characteristic curve of less than 60% for all RAMs.

**Meaning:**

This study suggests that the accuracy and prognostic performance of the simplified Geneva score and other validated RAMs to predict VTE is limited and their clinical usefulness is thus questionable.

## Teaser

This cohort study prospectively validates the simplified Geneva score, a risk assessment model for venous thromboembolism (VTE), and compares its performance with that of previously validated risk assessment models among Swiss inpatients.

## Introduction

Venous thromboembolism (VTE) represents one of the leading avoidable causes of death among hospitalized patients.^1^ Although particularly common among patients undergoing surgery,^2^ about 75% of hospital-acquired cases of VTE occur in nonsurgical patients.^3^ Pharmacologic thromboprophylaxis reduces the risk of VTE among selected medical inpatients.^4,5,6^ However, given the associated small increase in bleeding risk and the low baseline VTE incidence in the overall population of medical inpatients,^4,7^ its provision should be targeted to patients at increased risk of VTE.^4,7,8^

Although risk stratification in surgical patients is based mostly on the type of intervention,^2^ assessment of VTE risk among medical patients is more challenging and requires integration of various individual risk factors.^9,10^ To simplify and standardize VTE risk assessment among medical inpatients, risk assessment models (RAMs) such as the original Geneva score,^11^ the Padua score,^12^ or the IMPROVE (International Medical Prevention Registry on Venous Thromboembolism) score^13^ have been developed, and their use is encouraged by clinical guidelines.^7,8,14^ The practical usefulness of current RAMs is, however, limited by suboptimal sensitivities,^15^ nonuniform cutoff values to define risk groups,^13,16^ or a large number of variables.^11^ With the aim of developing a more usable RAM, the simplified Geneva score has been derived.^17^ A retrospective external validation study showed good discrimination and calibration of the simplified Geneva score^18^; however, prospective validation is currently lacking. In addition, the comparative performance of validated RAMs has not been examined prospectively. Using data from a prospective multicenter cohort of medical inpatients, we aimed to validate the simplified Geneva score and to perform a head-to-head comparison of its prognostic performance with previously validated RAMs.

## Methods

### Study Design and Setting

RISE (Risk Stratification for Hospital-Acquired Thromboembolism in Medical Patients) is a multicenter prospective cohort study of medical patients admitted to 3 Swiss tertiary care hospitals from June 18, 2020, to January 4, 2022 (ClinicalTrial.gov NCT04439383). The methods have been previously described.^19^ Reporting conforms to the Transparent Reporting of a Multivariable Prediction Model for Individual Prognosis or Diagnosis (TRIPOD) reporting guideline and checklist for prediction model validation.^20^ The study was conducted in accordance with all applicable legal and regulatory requirements. Authorization was granted from the responsible ethics committees (Kantonale Ethikkommission Bern, Commission cantonale d’éthique de la recherche sur l’être humain CER-VD, and Commission Cantonale d’Ethique de la Recherche sur l’être humain [CCER]), and written informed consent was obtained from all study participants.

### Population

Consecutive adults hospitalized in general internal medicine were screened on weekdays, and eligible patients were enrolled within 72 hours of admission. We included acutely ill patients aged 18 years or older who were admitted for hospitalization for more than 24 hours. Exclusion criteria were indication for therapeutic anticoagulation, estimated life expectancy less than 30 days, transfer from the intensive care unit or other wards, insufficient German or French language proficiency, prior enrollment in the study, and unwillingness to provide informed consent. For patients unable to consent due to mental illness or cognitive impairment, written consent was obtained from an authorized representative.

### VTE RAMs

At baseline, study personnel collected data on demographics, comorbidities, and VTE risk factors (Table 1; eMethods in Supplement 1). The simplified and original Geneva score, the IMPROVE score, and the Padua score were calculated and patients were categorized as high or low VTE risk according to each RAM (eTable 1 in Supplement 1).^11,12,13,17^ Treating physicians were not informed of the scores and the use of thromboprophylaxis was not influenced by the study. No RAM was implemented in order sets, but internal guidelines suggested to use the Padua score in 2 centers and the simplified Geneva score in 1 center to assess the indication for thromboprophylaxis. For patients at high risk of VTE, pharmacologic thromboprophylaxis was recommended, or nonpharmacologic prophylaxis for those at high bleeding risk.

**Table 1.** Baseline Patient Characteristics, Stratified by Low and High Risk of VTE According to the Simplified Geneva Score^a^

Characteristic	Patients, No. (%)			*P* value
	Total (N = 1352)	Low risk (n = 498)	High risk (n = 854)	
Age, median (IQR), y	67 (54-77)	56 (40-71)	71 (61-79)	<.001
Sex				
Female	590 (43.6)	219 (44.0)	371 (43.4)	.85
Male	762 (56.4)	279 (56.0)	483 (56.6)	
Items of the VTE risk assessment models				
Aged >60 y	846 (62.6)	185 (37.1)	661 (77.4)	<.001
Aged ≥70 y	588 (43.5)	130 (26.1)	458 (53.6)	<.001
BMI >30	269 (19.9)	62 (12.4)	207 (24.2)	<.001
Previous VTE^b^	88 (6.5)	0	88 (10.3)	<.001
Hypercoagulable state or thrombophilia^c^	12 (0.9)	2 (0.4)	10 (1.2)	.15
Active cancer^d^	263 (19.5)	22 (4.4)	241 (28.2)	<.001
Cardiac failure	134 (9.9)	4 (0.8)	130 (15.2)	<.001
Respiratory failure	237 (17.5)	8 (1.6)	229 (26.8)	<.001
Acute infection	581 (43.0)	81 (16.3)	500 (58.5)	<.001
Myeloproliferative syndrome	12 (0.9)	0	12 (1.4)	.008
Immobilization ≥3 d^e^	382 (28.3)	28 (5.6)	354 (41.5)	<.001
Immobilization ≥7 d^f^	110 (8.1)	12 (2.4)	98 (11.5)	<.001
Reduced mobility for ≥3 d^g^	485 (35.9)	96 (19.3)	389 (45.6)	<.001
Recent myocardial infarction (≤1 mo)	26 (1.9)	10 (2.0)	16 (1.9)	.86
Recent stroke (≤3 mo)	12 (0.9)	4 (0.8)	8 (0.9)	.80
Recent trauma (≤1 mo)	84 (6.2)	32 (6.4)	52 (6.1)	.80
Recent surgery (≤1 mo)	49 (3.6)	11 (2.2)	38 (4.4)	.03
Acute rheumatologic disease	54 (4.0)	7 (1.4)	47 (5.6)	<.001
Ongoing hormonal treatment	58 (4.3)	40 (8.0)	18 (2.1)	<.001
Lower extremity paralysis or paresis	28 (2.1)	4 (0.8)	24 (2.8)	.01
Stay in intensive or coronary care unit	0	0	0	NA
Nephrotic syndrome	7 (0.5)	2 (0.4)	5 (0.6)	.65
Recent travel^h^	36 (2.7)	12 (2.4)	24 (2.8)	.66
Chronic venous insufficiency	254 (18.8)	67 (13.4)	187 (21.9)	<.001
Pregnancy	4 (0.7)	4 (1.8)	0	.009
Dehydration	158 (11.7)	39 (7.8)	119 (13.9)	<.001
Other comorbidities				
Peripheral vascular disease	121 (8.9)	29 (5.8)	92 (10.8)	.001
Liver disease	98 (7.2)	50 (10.0)	48 (5.6)	.003
Dementia	54 (4.0)	11 (2.2)	43 (5.0)	.01
Cerebrovascular disease	116 (8.6)	26 (5.2)	90 (10.5)	<.001
Chronic obstructive pulmonary disease	169 (12.5)	32 (6.4)	137 (16.0)	<.001
Prior bleeding within last 3 mo	114 (8.4)	43 (8.6)	71 (8.3)	.84
Diabetes	297 (22.0)	105 (21.1)	192 (22.5)	.55
Laboratory findings				
Creatinine clearance <30 mL/min	118 (8.7)	25 (5.0)	93 (10.9)	<.001
Platelet count <50 cells × 10^3^/µL	36 (2.7)	15 (3.0)	21 (2.5)	.53
INR >1.5	18 (1.4)	12 (2.7)	6 (0.8)	.006
Hemoglobin, median (IQR), g/dL	12.8 (11.3-14.2)	13.2 (11.8-14.5)	12.7 (10.9-14.0)	<.001
Treatments at admission				
Aspirin	365 (27.0)	73 (14.6)	292 (34.2)	<.001
Other antiplatelet therapy^i^	95 (7.0)	22 (4.4)	73 (8.5)	.004
Dual antiplatelet therapy^j^	40 (3.0)	9 (1.8)	31 (3.6)	.06
NSAIDs	82 (6.1)	38 (7.6)	44 (5.2)	.07
VTE prophylaxis at baseline^k^	698 (51.6)	185 (37.1)	513 (60.0)	<.001
Pharmacological prophylaxis	687 (98.4)	183 (98.9)	504 (98.2)	NA
Mechanical prophylaxis	11 (1.6)	2 (1.1)	9 (1.8)	NA

Abbreviations: BMI, body mass index (calculated as weight in kilograms divided by height in meters squared); INR, international normalized ratio; NA, not available; NSAID, nonsteroid anti-inflammatory drug; VTE, venous thromboembolism.

SI conversion factors: To convert platelets to cells ×10^9^/L, multiply by 1.0; and hemoglobin to grams per liter, multiply by 10.0.

^a^ *P* values were calculated using the χ^2^ test, *t* test, or Wilcoxon rank sum test as appropriate. Data were missing for creatinine clearance (1 patient at low risk), platelet count (3 patients at low risk), INR (49 patients at low risk and 58 patients at high risk), and hemoglobin (2 patients at low risk).

^b^ Defined as prior deep vein thrombosis or pulmonary embolism.

^c^ Defined as antithrombin deficiency, activated protein C resistance, protein C or protein S deficiency, factor V Leiden, G20210A prothrombin mutation, or antiphospholipid syndrome.

^d^ Defined as metastatic cancer or cancer treated with radiotherapy, chemotherapy, immunotherapy, or cancer surgery within last 6 months.

^e^ Defined as complete bed rest or inability to walk for more than 30 minutes per day for 3 or more days.

^f^ Defined as confinement to chair or bed with or without bathroom privileges for 7 or more days immediately prior to and during hospital admission.

^g^ Defined as anticipated bed rest with or without bathroom privileges for 3 or more days.

^h^ Defined as more than 6 hours within the last 7 days.

^i^ Such as clopidogrel, ticagrelor, and prasugrel.

^j^ Defined as aspirin plus other antiplatelet therapy.

^k^ None of the patients received thromboprophylaxis with intermediate-dose low-molecular-weight heparin.

### Outcome

The primary outcome was symptomatic, objectively confirmed fatal and nonfatal VTE, including pulmonary embolism as well as distal and proximal deep vein thrombosis of the lower and upper extremity within 90 days of admission (eMethods in Supplement 1). To exclude preexisting VTE, we did not consider VTE diagnosed within 48 hours of admission.^21^ To assess VTE outcomes, study personnel blinded to RAM scores conducted follow-up visits on the day prior to discharge or the day of discharge, and contacted participants, their contact persons, and/or primary care physicians by telephone 90 days after admission.^11,22^ In case of a VTE outcome, medical and radiologic reports were collected to assess the date, type, and circumstances of the event. For participants who died, the cause was recorded based on medical reports, death certificates, and autopsy reports, if available (eMethods in Supplement 1). All VTE outcomes and deaths were adjudicated by a committee of 3 independent clinical experts blinded to RAM scores.

### Statistical Analysis

The sample size was calculated to validate the simplified Geneva score for the prediction of hospital-acquired VTE. Assuming that 67% of patients would be categorized as high risk based on the simplified Geneva score, and assuming a 90-day VTE incidence of 2.8% among patients at high risk and 0.6% among patients at low risk based on a previous study,^17^ we determined that recruitment of 1308 patients would be required to detect an absolute risk difference of 2.2%, with a power of 80% at a 2-sided α of .05. To account for potential dropouts, we aimed to recruit 1350 participants.

Standard descriptive statistical tests were used to compare low and high VTE risk groups based on the simplified Geneva score. Time-to-event analyses with competing risk methods were used to assess the prognostic performance of the simplified Geneva score and the other RAMs and their association with VTE, with non-VTE death representing the competing risk, using a subdistribution hazard model of Fine and Gray.^23^ Subhazard ratios with 95% CIs were calculated, first unadjusted and then adjusted for pharmacologic thromboprophylaxis use as a time-varying covariate and for study site. Cumulative incidences of VTE among patients at low risk and patients at high risk were assessed using Kaplan-Meier curves, with calculation of *P* values based on log-rank tests. Sensitivity, specificity, and positive and negative predictive values and likelihood ratios were determined for each RAM. The area under the curve (AUC) was calculated to assess the discriminative power of each continuous score using time-dependent receiver operating characteristic curve analysis, considering censored data and competing events. Calibration was determined using the Hosmer-Lemeshow goodness-of-fit test; use of a calibration plot was not possible because 2 of the RAMs were derived empirically (ie, based on literature or clinical expertise) rather than data driven.^12,17,18^

Patients for whom therapeutic anticoagulation was started for reasons other than VTE during follow-up were censored in the main analysis. Patients who were lost to follow-up were censored at the last visit.

We performed a subgroup analysis of patients who did not receive pharmacologic thromboprophylaxis at any time during hospitalization, and a subgroup analysis stratified by antiplatelet treatment during hospitalization. In a sensitivity analysis, we investigated how different outcome scenarios among patients lost to follow-up would be associated with discriminative performance of RAMs. The scenarios included VTE occurring (1) in all patients lost to follow-up, (2) in patients at high risk, and 3) in patients at low risk only.

Stata, version 17 (StataCorp LLC), and R, version 4.2.2 (R Project for Statistical Computing), were used for all analyses. A 2-sided *P* < .05 was considered statistically significant.

## Results

Of 4205 patients screened, 1352 were included in the RISE cohort (eFigure in Supplement 1). The median age was 67 years (IQR, 54-77 years) (vs 76 years [IQR, 64-85 years] for those excluded), with 590 women (43.6%) and 762 men (56.4%) (Table 1). Overall, 263 patients (19.5%) had active cancer, and 88 (6.5%) had a previous VTE event. Immobilization for 3 days or more was recorded for 382 patients (28.3%), and 698 (51.6%) had a prescription for pharmacologic or mechanical VTE prophylaxis at baseline. The median length of stay was 7 days (IQR, 5-11 days). The proportion of patients in the low-risk category receiving thromboprophylaxis was 37.9% (174 of 459) based on the original Geneva score. The proportion of patients categorized as high risk of VTE was 63.2% (n = 854) based on the simplified Geneva score, slightly higher with the original Geneva score (66.1% [n = 893]), and lower with the Padua score (47.8% [n = 646]) and the IMPROVE score (29.8% [n = 403]) (eFigure in Supplement 1).

Of all 1352 study participants, 10 (0.7%) were lost to follow-up and 88 (6.5%) died during the 90-day follow-up period. Venous thromboembolism occurred in 28 patients (2.1%); 18 events were pulmonary embolism (no fatal pulmonary embolism), and 10 were deep vein thrombosis.

**Figure 1.**
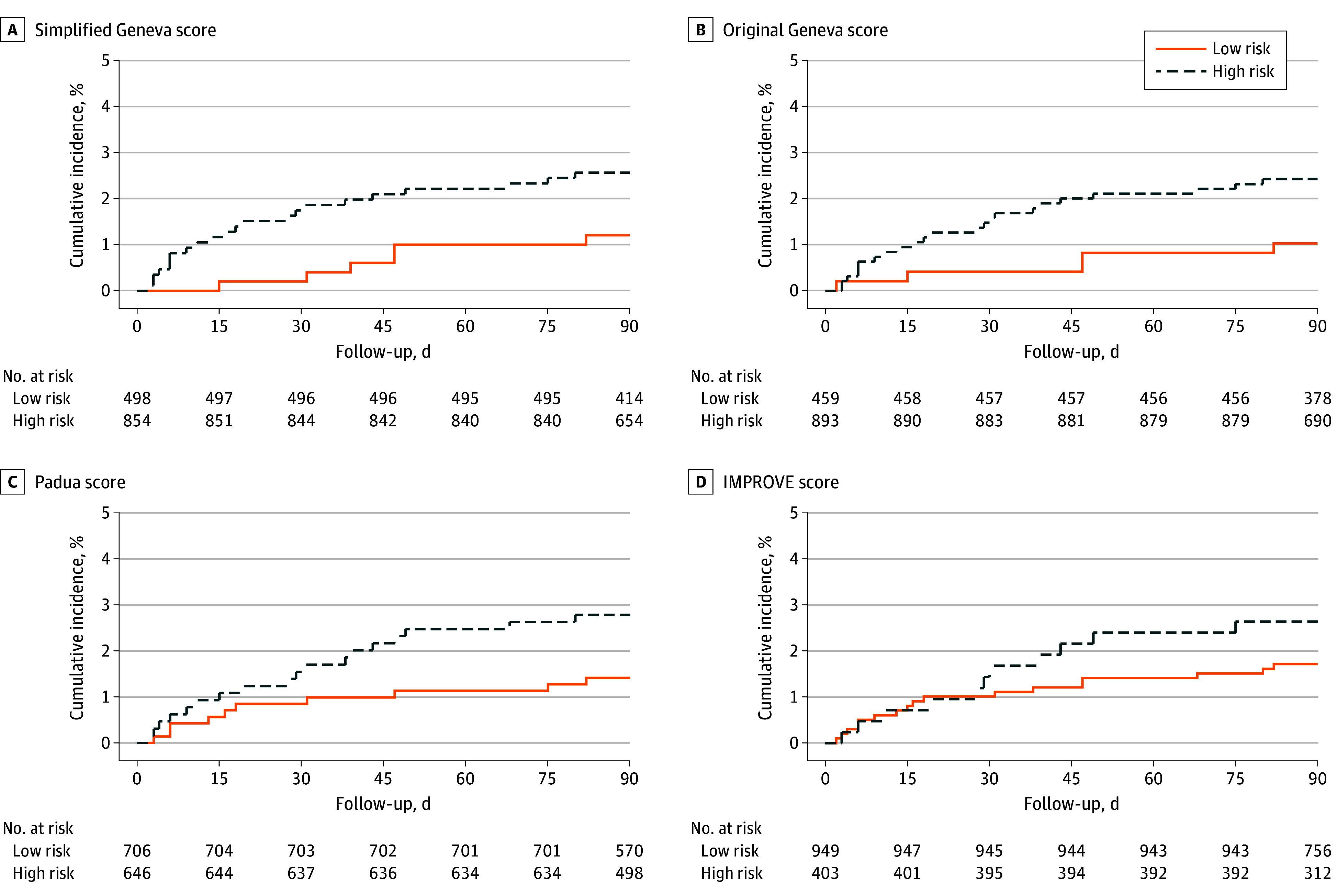
Kaplan-Meier Plot Showing the Cumulative Incidence of Venous Thromboembolism Among Patients at Low and High Risk A, Simplified Geneva risk score. The cumulative incidence was 1.2% (95% CI, 0.3%-2.2%) for patients at low risk and 2.6% (95% CI, 1.5%-3.6%) for patients at high risk (log-rank *P* = .09). B, Original Geneva risk score. The cumulative incidence was 1.1% (95% CI, 0.1%-2.0%) for patients at low risk and 2.6% (95% CI, 1.5%-3.6%) for patients at high risk (log-rank *P* = .07). C, Padua score. The cumulative incidence was 1.4% (95% CI, 0.5%-2.3%) for patients at low risk and 2.8% (95% CI, 1.5%-4.0%) for patients at high risk (log-rank *P* = .08). D, IMPROVE (International Medical Prevention Registry on Venous Thromboembolism) score. The cumulative incidence was 1.8% (95% CI, 0.9%-2.6%) for patients at low risk and 2.7% (95% CI, 1.1%-4.3%) for patients at high risk (log-rank *P* = .26).

According to the simplified Geneva score, VTE occurred in 2.6% (95% CI, 1.7%-3.9%) of patients at high risk (22 of 854) and 1.2% (95% CI, 0.6%-2.6%) of patients at low risk (6 of 498) (eTable 2 in Supplement 1). Similarly, VTE risk was 2.6% (95% CI, 1.7%-3.8%) (23 of 893) in the high-risk group and 1.1% (95% CI, 0.5%-2.5%) (5 of 459) in the low-risk group according to the original Geneva risk score, 2.8% (95% CI, 1.8%-4.4%) (18 of 646) in the high-risk group and 1.4% (95% CI, 0.8%-2.6%) (10 of 706) in the low-risk group based on the Padua score, and 2.7% (95% CI, 1.5%-4.8%) (11 of 403) in the high-risk group and 1.8% (95% CI, 1.1%-2.9%) (17 of 949) in the low-risk group based on the IMPROVE score. The 90-day cumulative incidence of VTE did not significantly differ between the low-risk and high-risk groups of the simplified Geneva score or in any risk groups based on the other RAMs (Figure 1).

Patients classified as high risk based on the simplified Geneva score did not have a statistically significantly increased VTE risk compared with those classified as low risk (adjusted subhazard ratio, 2.04 [95% CI, 0.83-5.05]; *P* = .12). Results were similar for the other 3 RAMs (Table 2). The simplified Geneva score showed a sensitivity of 78.6% (95% CI, 60.5%-89.8%) and a specificity of 37.2% (95% CI, 34.6%-39.8%) for the prediction of VTE (Table 3). Sensitivity was highest with the original Geneva score (82.1%; 95% CI, 64.4%-92.1%) and lowest with the IMPROVE score (39.3%; 95% CI, 23.6%-57.6%), while specificity was highest with the latter (70.4%; 95% CI, 67.9%-72.8%). The positive predictive value of the simplified Geneva score was 2.6% (95% CI, 1.7%-3.9%), while the negative predictive value was 98.8% (95% CI, 97.4%-99.4%); the positive likelihood ratio was 1.25 (95% CI, 1.03-1.52), and the negative likelihood ratio was 0.58 (95% CI, 0.28-1.18). Positive predictive values, negative predictive values, and positive and negative likelihood ratios of the other RAMs were similar.

**Table 2.** Risk of Hospital-Acquired Venous Thromboembolism in High-Risk vs Low-Risk Groups Based on Each Risk Assessment Model

Risk assessment model	Unadjusted SHR (95% CI)	*P* value	Adjusted SHR (95% CI)^a^	*P* value
Simplified Geneva score	2.16 (0.88-5.31)	.09	2.04 (0.83-5.05)	.12
Original Geneva score	2.38 (0.91-6.26)	.08	2.26 (0.86-5.98)	.10
Padua score	1.98 (0.91-4.29)	.08	2.03 (0.94-4.37)	.07
IMPROVE score	1.53 (0.72-3.26)	.27	1.52 (0.72-3.23)	.28

Abbreviations: IMPROVE, International Medical Prevention Registry on Venous Thromboembolism; SHR, subhazard ratio.

^a^ Adjusted for site and use of pharmacologic thromboprophylaxis as a time-varying covariate.

**Table 3.** Predictive Accuracy of Each Risk Assessment Model for Hospital-Acquired Venous Thromboembolism

Risk assessment model	% (95% CI)				LHR (95% CI)	
	Sensitivity	Specificity	PPV	NPV	Positive	Negative
Simplified Geneva score	78.6 (60.5-89.8)	37.2 (34.6-39.8)	2.6 (1.7-3.9)	98.8 (97.4-99.4)	1.25 (1.03-1.52)	0.58 (0.28-1.18)
Original Geneva score	82.1 (64.4-92.1)	34.3 (31.8-36.9)	2.6 (1.7-3.8)	98.9 (97.5-99.5)	1.25 (1.05-1.49)	0.52 (0.23-1.16)
Padua score	64.3 (45.8-79.3)	52.6 (49.9-55.2)	2.8 (1.8-4.4)	98.6 (97.4-99.2)	1.36 (1.02-1.80)	0.68 (0.41-1.12)
IMPROVE score	39.3 (23.6-57.6)	70.4 (67.9-72.8)	2.7 (1.5-4.8)	98.2 (97.1-98.9)	1.33 (0.83-2.12)	0.86 (0.64-1.16)

Abbreviations: IMPROVE, International Medical Prevention Registry on Venous Thromboembolism; LHR, likelihood ratio; NPV, negative predictive value; PPV, positive predictive value.

**Figure 2.**
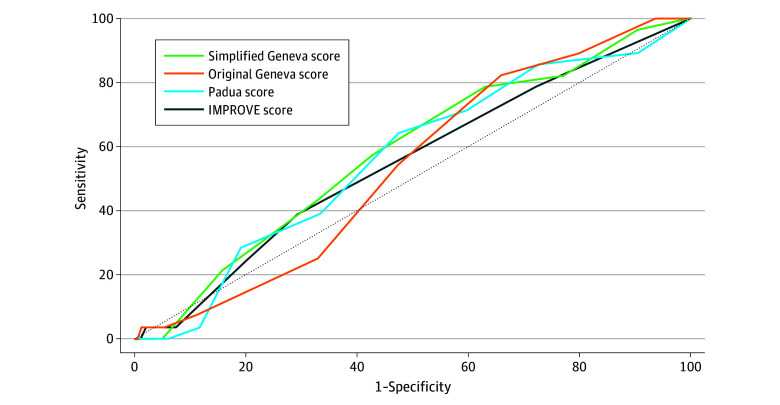
Receiver Operating Characteristic (ROC) Curves for Each Risk Assessment Model The area under the ROC curve was 58.1% for the simplified Geneva score (95% CI, 55.4%-60.7%), 53.8% (95% CI, 51.1%-56.5%) for the original Geneva score, 56.5% (95% CI, 53.7%-59.1%) for the Padua score, and 55.0% (95% CI, 52.3%-57.7%) for the IMPROVE (International Medical Prevention Registry on Venous Thromboembolism) score.

The discriminative performance was highest for the simplified Geneva score, with an AUC of 58.1% (95% CI, 55.4%-60.7%) and lowest for the original Geneva score, with an AUC of 53.8% (95% CI, 51.1%-56.5%), but overall poor for all RAMs (Figure 2; eTable 3 in Supplement 1). Calibration was acceptable for all RAMs (eTable 3 in Supplement 1).

In subgroup analyses of the 510 patients without pharmacologic thromboprophylaxis, VTE within 90 days occurred in 6 patients (1.2%) (eTable 4 in Supplement 1). The accuracy of the RAMs did not improve compared with the results in the overall population (eTable 5 in Supplement 1).

In an analysis stratified by antiplatelet treatment, VTE occurred in 9 of 420 patients (2.1%) with antiplatelet treatment and 19 of 932 patients (2.0%) without antiplatelet treatment (eTable 6 in Supplement 1). The accuracy of the RAMs remained similar irrespective of antiplatelet use (eTable 7 in Supplement 1). In a sensitivity analysis investigating different outcome scenarios in the 10 patients lost to follow-up, discriminative performance for all RAMs slightly increased when assuming that VTE occurred in patients at high risk only, with a maximum AUC of 61.9% for the Padua score (eTable 8 in Supplement 1).

## Discussion

In this prospective, multicenter cohort of medical inpatients, the simplified Geneva score showed a similarly poor prognostic accuracy and discriminative performance for predicting VTE compared with the original Geneva score, the Padua score, and the IMPROVE score. The cumulative incidence of VTE within 90 days for low-risk and high-risk categories of all 4 RAMs did not significantly differ. Overall, our results suggest that existing RAMs do not perform particularly well in identifying medical inpatients at risk for VTE.

We found no association between risk group and time to a first VTE event for all 4 RAMs. Although the overall incidence of VTE within 90 days was similar in our study compared with the derivation cohorts of the Geneva score, Padua score, and IMPROVE score, VTE incidence among those in the low-risk categories of our validation cohort was surprisingly high (1.1%-1.8% vs 0.3%-0.6% in the derivation cohorts of the RAMs)^11,12,13^ and above the 1% threshold that has been suggested for provision of thromboprophylaxis.^7^ A potential explanation for the comparatively high VTE incidence in the low-risk groups could possibly be associated with the differing proportions of patients with pharmacologic thromboprophylaxis.^11,17^ The proportion of patients in the low-risk category receiving thromboprophylaxis was lower in our cohort (37.9% [174 of 459] based on the original Geneva score)^24^ than in the derivation cohort (49%) of the original and simplified Geneva score,^17^ or other large cohorts.^1,10^

The sensitivities of the RAMs based on our study were lower than in previous cohorts.^11,25,26^ For example, sensitivity ranged from 73% to 90% (for the original and simplified Geneva scores, Padua score, and IMPROVE score) in a post hoc analysis from a Swiss prospective cohort, and from 74% to 92% (for the Caprini score, IMPROVE score, and Padua score) in a retrospective analysis from the French PREVENU (Prevention of Venous Thromboembolism Disease in Emergency Departments) study.^17,26^ Sensitivity is critical in RAMs to select patients for whom a preventive intervention (ie, thromboprophylaxis) can be safely forgone.^27^ However, specificity should also be considered: use of the simplified and original Geneva scores to target thromboprophylaxis prescription may result in overtreatment due to their low specificity and high sensitivity.

The discriminative performance for 90-day VTE was poor in our study, with an AUC of 53.8% to 58.1%. Although some previous validation studies (based on retrospective data or post hoc analyses of prospectively collected data) showed better discriminative performance (AUC >70%),^16,17^ poor results had also been reported in an external validation study of RAMs (including the Padua score and the IMPROVE score) using medical record data from Michigan hospitals,^28^ as well as the retrospective analysis of the PREVENU study.^26^

There are several potential explanations for the different results of our study and derivation or other validation studies of the Geneva score, Padua score, and IMPROVE score.^16,17^ First, VTE risk in low- and high-risk groups based on RAMs can be overestimated or underestimated by differing thromboprophylaxis use in the risk groups. Second, differences could be due to the definition of immobility, which differs between RAMs.^29^ Subjective estimation of mobility is inaccurate,^30^ and often surrogates such as the ability to go to the bathroom are used to quantify mobility.^11,16,17^ As mobility is a highly weighted item in all these RAMs, objective mobility measures (eg, from accelerometry) may improve estimation of VTE risk. Third, data of derivation studies and some validation studies have been collected more than 10 years ago,^11,13,16,18^ and inpatient care practices have changed within the last decade (eg, with shorter hospital stays, intensified in-hospital mobilization), with a direct association with VTE risk.^14^ Fourth, although our cohort is generally comparable with the population of the derivation studies (eTable 9 in Supplement 1), there may be unmeasured variations in characteristics associated with VTE risk. For example, approximately one-third of the patients in our cohort received antiplatelet therapy, while these data are not reported for previous derivation and validation studies.^11,12,13,16,17^ However, antiplatelet treatment did not have a relevant association with accuracy measures in our study. In addition, subsequent hospitalizations and subsequent use of thromboprophylaxis may be associated with 90-day VTE risk.

Given the overall limited accuracy and prognostic performance of all analyzed RAMs, our results cast doubts on their reliability to identify medical inpatients at risk of VTE for whom thromboprophylaxis is warranted. Even though guidelines, including those from the American College of Chest Physicians or the National Institute for Health and Care Excellence, encourage the use of RAMs to identify medical inpatients at high VTE risk,^7,14^ our results emphasize the need for more accurate risk prediction strategies, as already advocated by others.^8^ For example, it is unclear whether the use of objective mobility measures or artificial intelligence–based models could improve VTE risk prediction.^19,31^ In addition, the clinical benefit associated with applying RAMs is unclear.^8,25^ Except for a single randomized trial that showed a reduction in VTE rates with a computer-alert program incorporating the Kucher RAM,^32^ no prospective comparative study has, to our knowledge, demonstrated improved clinical or economic outcomes with the application of RAMs in clinical practice.^33^ The overall necessity of VTE risk stratification to implement targeted thromboprophylaxis may be questioned in light of the uncertain net clinical benefit associated with thromboprophylaxis for medical inpatients.^34,35^ Randomized clinical trials conducted more than 15 years ago showed up to 63% reductions in VTE with pharmacologic thromboprophylaxis compared with placebo, although the results were mainly due to a reduced risk of asymptomatic VTE of unclear clinical relevance.^6,36,37^ The recently published SYMPTOMS (Systematic Elderly Medical Patients Thromboprophylaxis: Efficacy on Symptomatic Outcomes) trial did not show significant differences in symptomatic VTE at 30 days in more than 2500 older medical inpatients randomized to enoxaparin or placebo, albeit the trial was underpowered due to premature termination.^38^ In addition, thromboprophylaxis does not reduce mortality in medical inpatients,^5^ but may be associated with a small increase in bleeding risk based on results of a meta-analysis,^4^ although we did not find such an association in data from our cohort.^39^

### Limitations

Our study has some limitations. First, results may have been affected by thromboprophylaxis use, and unadjusted accuracy measures are therefore difficult to interpret. To address this limitation, we conducted a subgroup analysis among patients without thromboprophylaxis, but the size of the subpopulation was small and thus the analysis was underpowered. Thromboprophylaxis was not assigned at random, which may have had a negative association with measures of accuracy and discrimination due to lower actual VTE rates among patients at high risk for VTE. However, the potential for this bias is reduced by the relevant proportion of underuse of thromboprophylaxis for patients at high risk and overuse for patients at low risk, as previously demonstrated in our cohort.^11,24,40^ In addition, all 4 RAMs were derived in populations of patients with or without thromboprophylaxis, and withholding thromboprophylaxis to perform a derivation or validation study would be unethical. Second, the number of VTE events was low, with large 95% CIs around the estimates. Even though differences in VTE risk between low-risk and high-risk groups were not statistically significant, they may still be clinically relevant. Third, given that patients were recruited from Swiss university hospitals, our results may not be generalizable to health care settings outside of high-income countries or White populations. Fourth, patients at high risk may have been underrepresented in our cohort, given that patients screened but excluded were older than those included, although this may be mostly explained by exclusion of populations for whom RAMs are irrelevant (eg, those receiving therapeutic anticoagulation or with a life expectancy <30 days). Fifth, we did not use specific criteria to define recurrent deep vein thrombosis.^41^ However, only 1 deep vein thrombosis event occurred in a patient with prior VTE.

## Conclusions

To our knowledge, this cohort study provides the first prospective head-to-head comparison of validated RAMs. The easy-to-use simplified Geneva score showed similarly poor performance in predicting the risk for hospital-acquired VTE among medical inpatients compared with other validated RAMs. Overall, accuracy and prognostic performance of all analyzed RAMs were limited, questioning their clinical usefulness. More accurate strategies to predict VTE risk among medical inpatients as well as randomized studies evaluating the effect of risk assessment strategies are needed.

## Notes

Supplement 1. **eMethods.** Variable Definitions **eFigure.** Flow Chart **eTable 1.** Venous Thromboembolism Risk Assessment Models **eTable 2.** Venous Thromboembolism Events in Low- and High-Risk Patients According to the Four Risk Assessment Models **eTable 3.** Discrimination and Goodness of Fit of Each Risk Assessment Model to Predict Hospital-Acquired Venous Thromboembolism **eTable 4.** Venous Thromboembolism Events in Patients Without Pharmacological Thromboprophylaxis According to the Four Risk Assessment Models **eTable 5.** Predictive Accuracy of Risk Assessment Models for Hospital-Acquired Venous Thromboembolism in Patients Without Pharmacological Thromboprophylaxis **eTable 6.** Venous Thromboembolism Events in Low- and High-Risk Patients According to the Four Risk Assessment Models, Stratified by Antiplatelet Treatment During Hospitalization **eTable 7.** Predictive Accuracy of Risk Assessment Models for Hospital-Acquired Venous Thromboembolism, Stratified by Antiplatelet Treatment During Hospitalization **eTable 8.** Sensitivity Analysis Investigating the Discriminative Performance of Risk Assessment Models With Different Outcome Scenarios Among Patients Lost to Follow-up **eTable 9.** Demographics, Predictors and Outcomes of Participants in the RISE Study and the Derivation Cohorts of the Four Risk Assessment Models
eReferences.

Supplement 2.
Data Sharing Statement
