# Supplementary material for: Risk Assessment Models for Venous Thromboembolism in Medical Inpatients
Source: JAMA Netw Open. 2024 May 10;7(5):e249980. doi: 10.1001/jamanetworkopen.2024.9980 (PMC11087835; doi:10.1001/jamanetworkopen.2024.9980)
Supplement: Supplement 1. — eMethods. Variable Definitions eFigure. Flow Chart eTable 1. Venous Thromboembolism Risk Assessment Models eTable 2. Venous Thromboembolism Events in Low- and High-Risk Patients According to the Four Risk Assessment Models eTable 3. Discrimination and Goodness of Fit of Each Risk Assessment Model to Predict Hospital-Acquired Venous Thromboembolism eTable 4. Venous Thromboembolism Events in Patients Without Pharmacological Thromboprophylaxis According to the Four Risk Assessment Models eTable 5. Predictive Accuracy of Risk Assessment Models for Hospital-Acquired Venous Thromboembolism in Patients Without Pharmacological Thromboprophylaxis eTable 6. Venous Thromboembolism Events in Low- and High-Risk Patients According to the Four Risk Assessment Models, Stratified by Antiplatelet Treatment During Hospitalization eTable 7. Predictive Accuracy of Risk Assessment Models for Hospital-Acquired Venous Thromboembolism, Stratified by Antiplatelet Treatment During Hospitalization eTable 8. Sensitivity Analysis Investigating the Discriminative Performance of Risk Assessment Models With Different Outcome Scenarios Among Patients Lost to Follow-up eTable 9. Demographics, Predictors and Outcomes of Participants in the RISE Study and the Derivation Cohorts of the Four Risk Assessment Models eReferences. [file jamanetwopen-e249980-s001.pdf]

Supplementary Online Content

Häfliger E, Kopp B, Darbellay Farhoumand P, et al. Risk assessment models for venous thromboembolism in medical inpatients. *JAMA Netw Open.* 2024;7(5):e249980. doi:10.1001/jamanetworkopen.2024.9980

eMethods. Variable Definitions
eFigure. Flow Chart
eTable 1. Venous Thromboembolism Risk Assessment Models
eTable 2. Venous Thromboembolism Events in Low- and High-Risk Patients According to the Four Risk Assessment Models
eTable 3. Discrimination and Goodness of Fit of Each Risk Assessment Model to Predict Hospital-Acquired Venous Thromboembolism
eTable 4. Venous Thromboembolism Events in Patients Without Pharmacological Thromboprophylaxis According to the Four Risk Assessment Models
eTable 5. Predictive Accuracy of Risk Assessment Models for Hospital-Acquired Venous Thromboembolism in Patients Without Pharmacological Thromboprophylaxis
eTable 6. Venous Thromboembolism Events in Low- and High-Risk Patients According to the Four Risk Assessment Models, Stratified by Antiplatelet Treatment During Hospitalization
eTable 7. Predictive Accuracy of Risk Assessment Models for Hospital-Acquired Venous Thromboembolism, Stratified by Antiplatelet Treatment During Hospitalization
eTable 8. Sensitivity Analysis Investigating the Discriminative Performance of Risk Assessment Models With Different Outcome Scenarios Among Patients Lost to Follow-up
eTable 9. Demographics, Predictors and Outcomes of Participants in the RISE Study and the Derivation Cohorts of the Four Risk Assessment Models
eReferences.

This supplementary material has been provided by the authors to give readers additional information about their work.

© 2024 Häfliger E et al. *JAMA Network Open.*

eMethods. Variable Definitions

Variable	Definition
Previous venous thromboembolism	prior deep vein thrombosis or pulmonary embolism
Hypercoagulable state	any diagnosis of factor V Leiden, antithrombin deficiency, activated protein C resistance, protein C or protein S deficiency, G20210A prothrombin-mutation, or antiphospholipid syndrome
Cancer	metastatic cancer, cancer treated with radiotherapy, chemotherapy, immunotherapy, or surgery within the past 6 months
Immobilization	if the patient was being confined to chair or bed with or without bathroom privileges for ≥7 days immediately prior to and during hospital admission for the IMPROVE score[1]<br>in case of complete bedrest or inability to walk for 30 minutes per day during ≥3 days for the original and simplified Geneva score[1,2]
Reduced mobility	anticipated bed rest with or without bathroom privileges for ≥3 days for the Padua score[3]
Recent travel	any travel >6 hours within the last 7 days
Objective pulmonary embolism	new intraluminal filling defect on computed tomography pulmonary angiography (CTPA) or pulmonary angiography; a high probability ventilation/perfusion lung scan; a new pulmonary embolism on autopsy, or an objectively confirmed proximal deep vein thrombosis with clinical signs and symptoms of pulmonary embolism, as described previously.[4]
Objective deep vein thrombosis	non-compressible venous segment on compression ultrasonography or an intraluminal filling defect on contrast venography.[4] Diagnostic criteria for iliac and caval deep vein thrombosis additionally included abnormal duplex flow patterns compatible with thrombosis or an intraluminal filling defect on computed tomography or magnetic resonance imaging venography.[5]
Pulmonary embolism-related death	Death was considered PE-related in the following situations: a) autopsy-confirmed PE in the absence of another more likely cause of death, b) objectively confirmed PE within the last 48 hours before death in the absence of another more likely cause of death, or c) PE not objectively confirmed, but most likely the main cause of death.[6]

© 2024 Häfliger E et al. *JAMA Network Open.*

eFigure. Flow Chart

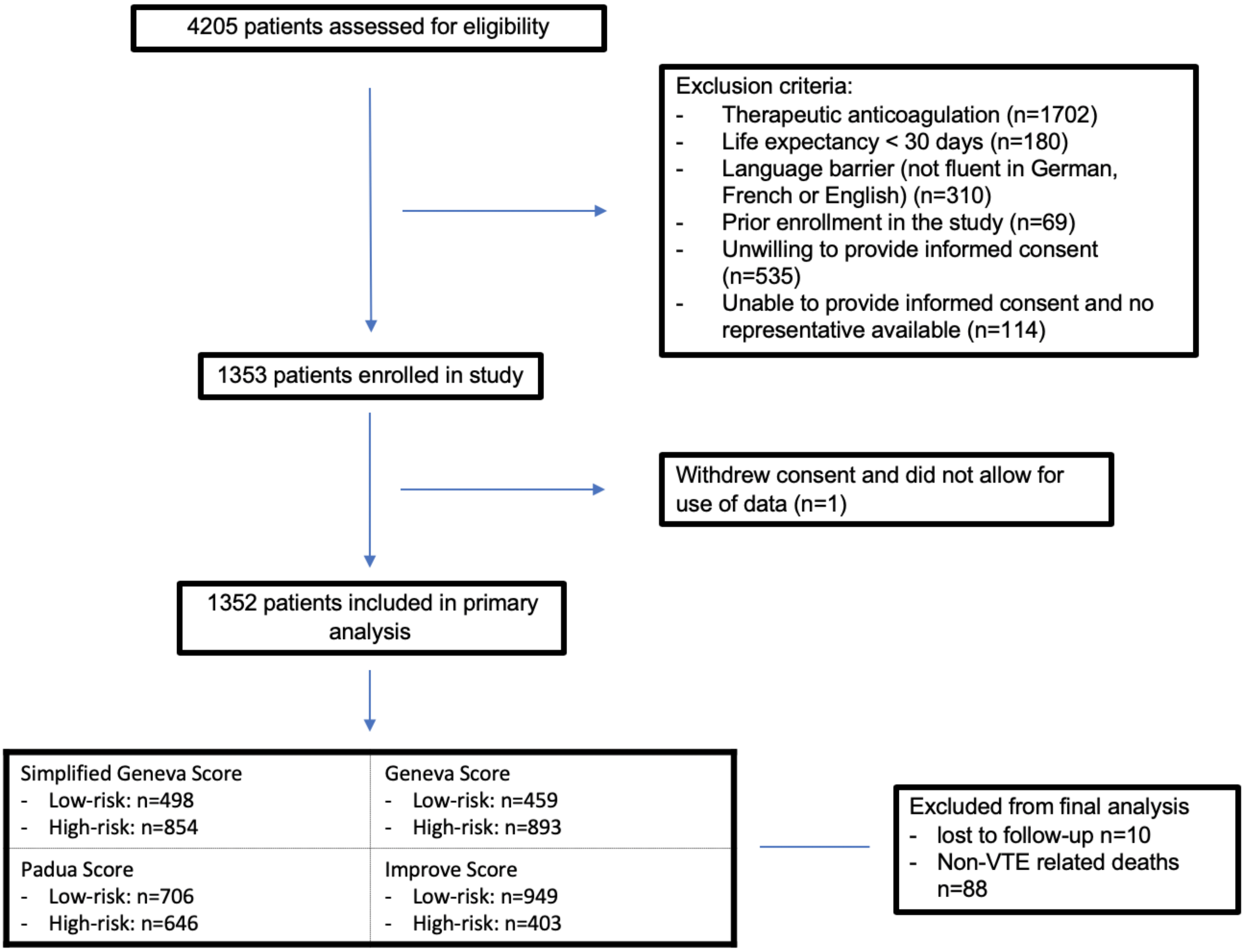

Some of the excluded participants had multiple exclusion criteria

© 2024 Häfliger E et al. *JAMA Network Open.*

eTable 1. Venous Thromboembolism Risk Assessment Models

	Points			
**Score Items**	**Simplified Geneva score**[1,2]	**Original Geneva score**[7]	**Padua score**[3]	**IMPROVE score**[8,9]
Previous VTE	3	2	3	3
Hypercoagulable state	2	2	3	2
Cancer[a]	2	2	3	2
Myeloproliferative syndrome		2	-	-
Cardiac failure	2	2	1	-
Respiratory failure		2		-
Acute infection	2	2	1	-
Acute rheumatologic disorder		2		-
Immobilization[b]	2	1	-	1
Reduced mobility[c]	-	-	3	-
Lower limb paralysis or paresis	-	-	-	2
Age > 60 years	1	1	-	1
Age > 70 years	-	-	1	-
Body mass index > 30 kg/m^2	1	1	1	-
Recent stroke[d]	1	2	1	-
Recent myocardial infarction[e]		2		-
Nephrotic syndrome	-	2	-	-
Hormonal treatment	-	1	1	-
Recent travel (> 6 hours)[f]	-	1	-	-
Chronic venous insufficiency	-	1	-	-
Pregnancy	-	1	-	-
Dehydration	-	1	-	-
Recent trauma or surgery (<1 month)	-	-	2	-
Stay in intensive or coronary care unit	-	-	-	1
**Cut-offs**[1,8,10]				
Low VTE risk	0-2	0-2	0-3	0-1
High VTE risk	≥3	≥3	≥4	≥2

Abbreviation: VTE, venous thromboembolism.
[a] Defined as metastatic cancer, or cancer treated with radiotherapy, chemotherapy, immunotherapy, or cancer surgery within last 6 months; for the IMPROVE score, cancer was defined as any cancer within the last 5 years
[b] The definition of immobilization was met if the patient was being confined to bed or chair with or without bathroom privileges for ≥7 days immediately prior to and during hospital admission for the IMPROVE score; or in case of complete bedrest or inability to walk for >30min per day for ≥3 days for the simplified and original Geneva score
[c] Reduced mobility was defined as anticipated bed rest with bathroom privileges for ≥3 days for the Padua score
[d] Defined as stroke < 3 months ago for the original Geneva score; or any recent stroke for the Padua and simplified Geneva score
[e] Defined as recent myocardial infarction (< 4 weeks ago); or any recent myocardial infarction for the Padua and simplified Geneva score
[f] Within last 7 days

© 2024 Häfliger E et al. *JAMA Network Open.*

eTable 2. Venous Thromboembolism Events in Low- and High-Risk Patients According to the Four Risk Assessment Models

	Low risk		High risk		All patients	
**Risk assessment model**	**n/ N[a]**	**% (95 % CI)**	**n/ N[a]**	**% (95% CI)**	**n/ N[a]**	**% (95% CI)**
Simplified Geneva Score	6/498	1.20 (0.55 – 2.60)	22/854	2.58 (1.71 – 3.87)	28/1352	2.07 (1.44 – 2.98)
Geneva Score	5/459	1.09 (0.47 – 2.52)	23/893	2.58 (1.72 – 3.84)	28/1352	2.07 (1.44 – 2.98)
Padua Score	10/706	1.42 (0.77 – 2.59)	18/646	2.79 (1.77 – 4.36)	28/1352	2.07 (1.44 – 2.98)
IMPROVE Score	17/949	1.79 (1.12 – 2.85)	11/403	2.73 (1.53 – 4.82)	28/1352	2.07 (1.44 – 2.98)

Abbreviations: CI, confidence interval
[a] Number of VTE (n) and overall number of participants in risk group (N), with corresponding proportion of VTE events with its Wilson confidence interval

© 2024 Häfliger E et al. *JAMA Network Open.*

eTable 3. Discrimination and Goodness of Fit of Each Risk Assessment Model to Predict Hospital-Acquired Venous Thromboembolism

Risk assessment model	Time-dependent AUC (95% CI)	p-value Hosmer-Lemeshow
Simplified Geneva score	58.1 (55.4 – 60.7)	0.36
Geneva score	53.8 (51.1 – 56.5)	0.15
Padua score	56.5 (53.7 – 59.1)	0.08
IMPROVE score	55.0 (52.3 – 57.7)	0.29

Abbreviations: AUC, area under the receiver operating characteristic curve; CI, confidence interval

© 2024 Häfliger E et al. *JAMA Network Open.*

eTable 4. Venous Thromboembolism Events in Patients Without Pharmacological Thromboprophylaxis According to the Four Risk Assessment Models

	Low risk		High risk		All patients	
**Risk assessment model**	**n/ N[a]**	**% (95 % CI)**	**n/ N[a]**	**% (95% CI)**	**n/ N[a]**	**% (95% CI)**
Simplified Geneva Score	3/261	1.15 (0.39 – 3.32)	3/249	1.2 (0.41 – 3.48)	6/510	1.18 (0.54 – 2.54)
Geneva Score	3/239	1.26 (0.43 – 3.62)	3/271	1.11 (0.38 – 3.2)	6/510	1.18 (0.54 – 2.54)
Padua Score	2/342	0.58 (0.16 – 2.11)	4/168	2.38 (0.93 – 5.96)	6/510	1.18 (0.54 – 2.54)
IMPROVE Score	4/396	1.01 (0.39 – 2.57)	2/114	1.75 (0.48 – 6.17)	6/510	1.18 (0.54 – 2.54)

Abbreviations: CI, confidence interval

[a] Number of VTE (n) and overall number of participants without thromboprophylaxis in risk group (N), with corresponding proportion of VTE events and its Wilson confidence interval

© 2024 Häfliger E et al. *JAMA Network Open.*

eTable 5. Predictive Accuracy of Risk Assessment Models for Hospital-Acquired Venous Thromboembolism in Patients Without Pharmacological Thromboprophylaxis

Risk assessment model	Sensitivity (%)	Specificity (%)	PPV (%)	NPV (%)	Positive LHR	Negative LHR
	(95% CI)					
Simplified Geneva score	50<br>(19 – 81)	51<br>(47 – 56)	1.2<br>(0.4 – 3.5)	99<br>(97 – 99)	1<br>(0.5 – 2.3)	1<br>(0.4 – 2.2)
Geneva score	50<br>(19 – 81)	47<br>(43 – 51)	1.1<br>(0.4 – 3.2)	99<br>(96 – 99)	0.9<br>(0.4 – 2.1)	1.1<br>(0.5 – 2.4)
Padua score	67<br>(30 – 90)	68<br>(63 – 71)	2.4<br>(0.9 – 6)	99<br>(98 – 99)	2.1<br>(1.2 – 3.7)	0.5<br>(0.2 – 1.5)
IMPROVE score	33<br>(10 – 70)	78<br>(74– 80)	1.8<br>(0.5 – 6)	99<br>(97 – 99)	1.5<br>(0.5 – 4.7)	0.9<br>(0.5 – 1.5)

Abbreviations: PPV, positive predictive value; NPV, negative predictive value; LHR, likelihood ratio; CI, confidence interval

© 2024 Häfliger E et al. *JAMA Network Open.*

eTable 6. Venous Thromboembolism Events in Low- and High-Risk Patients According to the Four Risk Assessment Models, Stratified by Antiplatelet Treatment During Hospitalization

Risk assessment model	Aspirin or other antiplatelet therapy	Low risk		High risk		All patients	
		n/ N[a]	% (95 % CI)	n/ N[a]	% (95% CI)	n/ N[a]	% (95% CI)
Simplified Geneva Score	yes	0/86	0.00 (0.00 - 4.28)	9/334	2.69 (1.42 - 5.04)	9/420	2.14 (1.13 -4.02)
	no	6/412	1.46 (0.67 - 3.14)	13/520	2.50 (1.47 - 4.23)	19/932	2.04 (1.31 - 3.16)
Geneva Score	yes	1/89	1.12 (0.20 - 6.09)	8/331	2.42 (1.23 - 4.70)	9/420	2.14 (1.13 -4.02)
	no	4/370	1.08 (0.42 - 2.75)	15/562	2.67 (1.62 - 4.36)	19/932	2.04 (1.31 - 3.16
Padua Score	yes	4/199	2.01 (0.78 - 5.05)	5/221	2.26 (0.97 - 5.19)	9/420	2.14 (1.13 -4.02)
	no	6/507	1.18 (0.54 - 2.56)	13/425	3.06 (1.80 - 5.16)	19/932	2.04 (1.31 - 3.16
IMPROVE Score	yes	5/288	1.74 (0.74 - 4.00)	4/132	3.03 (1.18 - 7.53)	9/420	2.14 (1.13 -4.02)
	no	12/661	1.82 (1.04 - 3.15)	7/271	2.58 (1.26 - 5.23)	19/932	2.04 (1.31 - 3.16

Abbreviations: CI, confidence interval.
[a] Number of VTE (n) and overall number of participants in risk group (N), with corresponding proportion of VTE events and its Wilson confidence interval

© 2024 Häfliger E et al. *JAMA Network Open.*

eTable 7. Predictive Accuracy of Risk Assessment Models for Hospital-Acquired Venous Thromboembolism, Stratified by Antiplatelet Treatment During Hospitalization

Risk assessment model	Aspirin or other antiplatelet therapy	Sensitivity (%)	Specificity (%)	PPV (%)	NPV (%)	Positive LHR	Negative LHR
		(95% CI)					
Simplified Geneva score	yes	100<br>(70 – 100)	21<br>(17 – 25)	2.7<br>(1.4 – 5.0)	100<br>(96 – 100)	1.3<br>(1.2 – 1.3)	0.0<br>(. - .)
	no	68<br>(46 - 85)	45<br>(41 - 48)	2.5<br>(1.5 - 4.2)	99<br>(97 - 99)	1.2<br>(0.9 - 1.7)	0.7<br>(0.4 - 1.4)
Geneva score	yes	89<br>(57 – 98)	21<br>(18 – 26)	2.4<br>(1.2 – 4.7)	99<br>(94 – 100)	1.1<br>(0.9 – 1.4)	0.5<br>(0.1 – 3.3)
	no	79<br>(57 – 92)	40<br>(37 – 43)	2.7<br>(1.6 – 4.4)	99<br>(97 – 100)	1.3<br>(1.0 – 1.7)	0.5<br>(0.2 – 1.3)
Padua score	yes	56<br>(27 – 81)	47<br>(43 – 52)	2.3<br>(1.0 – 5.2)	98<br>(95 - 99)	1.1<br>(0.6 - 1.9)	0.9<br>(0.5 – 2.0)
	no	68<br>(46 – 85)	55<br>(52 – 58)	3.1<br>(1.8 – 5.2)	99<br>(97 – 100)	1.5<br>(1.1 – 2.1)	0.6<br>(0.3 – 1.1)
IMPROVE score	yes	44<br>(19 - 73)	69<br>(64 - 73)	3.0<br>(1.2 -7.5)	98<br>(96 - 99)	1.4<br>(0.7 - 3.0)	0.8<br>(0.5 - 1.5)
	no	37<br>(19 – 59)	71<br>(68 – 734)	2.6<br>(1.3 – 5.2)	98<br>(97 - 99)	1.3<br>(0.7 - 2.3)	0.9<br>(0.6 - 1.3)

Abbreviations: PPV, positive predictive value; NPV, negative predictive value; LHR, likelihood ratio; CI, confidence interval

© 2024 Häfliger E et al. *JAMA Network Open.*

eTable 8. Sensitivity Analysis Investigating the Discriminative Performance of Risk Assessment Models With Different Outcome Scenarios Among Patients Lost to Follow-up

Risk assessment model	Original AUC<br>%	High risk / all lost to follow-up<br>n/N	AUC if all lost to follow-up had VTE<br>%	AUC if high-risk only had VTE (best case scenario)<br>%	AUC if low-risk only had VTE (worst case scenario)<br>%
Simplified Geneva Score	58.1	9 / 10	58.9	60.1	56.6
Geneva Score	53.8	9 / 10	56.3	57.1	52.9
Padua Score	56.5	6 / 10	57.2	61.9	51.7
IMPROVE Score	55.0	4 / 10	54.6	59.0	50.8

Abbreviations: AUC, area under the receiver operating characteristic curve; VTE, venous thromboembolism

© 2024 Häfliger E et al. *JAMA Network Open.*

eTable 9. Demographics, Predictors and Outcomes of Participants in the RISE Study and the Derivation Cohorts of the Four Risk Assessment Models

Cohort characteristics	RISE (N=1352)	Original and Simplified Geneva Score[1,2,7] (N=1478)	Padua Score[3] (N=1180)	IMPROVE Score[8,11] (N=15,156)
Age (years), median (IQR)	67 [54, 77]	64.8 (SD 16.9)	NA	68 [52, 79]
Sex				
- Female	590 (44)	700 (47)	625 (53)	NA (50)
- Male	762 (56)	778 (53)	555 (47)	NA (50)
**Items of the VTE risk assessment models**				
Age > 60 years	846 (63)	960 (65)	NA	9646 (64)
Age ≥ 70 years	588 (43)	651 (44)	NA	NA
BMI > 30 (kg/m^2)	269 (20)	219 (15)	NA	NA
Previous VTE[a]	88 (6.5)	121 (8.2)	46 (3.9)	551 (4)
Hypercoagulable state/thrombophilia[b]	12 (0.9)	9 (0.61)	3 (0.25)	42 (0.3)
Active cancer[c]	263 (19)	376 (25)	234 (20)	1735 (12)
Cardiac failure	134 (10)	177 (12)	254 (22)	NA (11)
Respiratory failure	237 (18)	353 (24)		NA (19)
Acute infection	581 (43)	444 (30)	NA	NA (32)
Myeloproliferative syndrome	12 (0.9)	31 (2.1)	NA	NA
Immobilization ≥ 3 days[d]	382 (28)	551 (37)	NA	NA (33)
Immobilization ≥ 7 days[e]	110 (8.1)	NA	NA	2846 (19)
Reduced mobility for ≥ 3 days[f]	485 (36)	NA	272 (23)	NA
Recent myocardial infarction (≤1 month)	26 (1.9)	30 (2)	12 (1)	NA
Recent stroke (≤3 months)	12 (0.9)	31 (2.1)		NA (6)
Recent trauma (≤1 month)	84 (6.2)	94 (6.4)	31 (2.6)	NA
Recent surgery (≤1 month)	49 (3.6)			NA
Acute rheumatologic disease	54 (4)	60 (4.1)	NA	NA (7)
Ongoing hormonal treatment	58 (4.3)	69 (4.7)	13 (1.1)	252 (2)
Lower extremity paralysis/paresis	28 (2.1)	NA	NA	239 (2)
Stay in intensive or coronary care unit	0	NA	NA	741 (5)
Nephrotic syndrome	7 (0.52)	24 (1.6)	NA	NA
Recent travel[g]	36 (2.7)	50 (3.4)	NA	NA
Chronic venous insufficiency	254 (19)	97 (6.6)	NA	NA
Pregnancy	4 (0.3)	3 (0.2)	NA	NA
Dehydration	158 (12)	168 (11)	NA	NA
**Thromboprophylaxis**				
Thromboprophylaxis in low-risk patients (n/N[h])	NA	245/516 (48)	52/711 (7.3)	NA
Thromboprophylaxis in high-risk patients (n/N[h])	NA	596/962 (62)	186/469 (40)	NA
Overall thromboprophylaxis during hospitalization (n/N[i])	842/1352 (62)	865/1478 (59)[k]	238/1180 (20)	6864/15156 (45)
**Outcome**				
90-day VTE	28 (2.1)	30 (2.0)	37 (3.1)	143 (1)

Numbers are presented as n (%), unless indicated otherwise. Abbreviations: BMI, body mass index; CVI, cerebrovascular insult; DAPT, dual antiplatelet therapy; INR, International Normalized Ratio; IQR, interquartile range; LMWH, low molecular weight heparine; NA, not available/applicable; NSAID, non-steroid anti-inflammatory drug; NSTEMI, Non-ST-elevation myocardial infarction; SD, standard deviation; STEMI, ST-Elevation Myocardial Infarction; TIA, transient ischemic attack; TPX, thromboprophylaxis; VTE, venous thromboembolism

[a] Defined as prior deep vein thrombosis or pulmonary embolism
[b] Defined as antithrombin deficiency, activated protein C resistance, protein C or protein S deficiency, factor V Leiden, G20210A prothrombin-mutation, or antiphospholipid syndrome
[c] Defined as metastatic cancer, or cancer treated with radiotherapy, chemotherapy, immunotherapy, or cancer surgery within last 6 months
[d] Defined as complete bedrest or inability to walk for >30 minutes per day for ≥3 days

© 2024 Häfliger E et al. *JAMA Network Open.*

[e] Defined as confinement to chair or bed with or without bathroom privileges for ≥7 days immediately prior to and during hospital admission
[f] Defined as anticipated bed rest with or without bathroom privileges for ≥3 days
[g] Defined as > 6 hours within the last 7 days
[h] Number of patients with thromboprophylaxis (n) among all participants in risk group (N), with corresponding proportion; not applicable for the RISE cohort, because this is depending on the RAM
[i] Number of patients with thromboprophylaxis (n) among all participants (N), with corresponding proportion
[k] thromboprophylaxis administered during the first 48h of hospitalization

© 2024 Häfliger E et al. *JAMA Network Open.*

eReferences.

1. Blondon M, Spirk D, Kucher N, et al. Comparative Performance of Clinical Risk Assessment Models for Hospital-Acquired Venous Thromboembolism in Medical Patients. *Thromb Haemost*. 2018;118(01):082-089. doi:10.1160/TH17-06-0403
2. Blondon M, Righini M, Nendaz M, et al. External validation of the simplified Geneva risk assessment model for hospital-associated venous thromboembolism in the Padua cohort. *Journal of Thrombosis and Haemostasis*. 2020;18(3):676-680. doi:10.1111/jth.14688
3. Barbar S, Noventa F, Rossetto V, et al. A risk assessment model for the identification of hospitalized medical patients at risk for venous thromboembolism: the Padua Prediction Score. *Journal of Thrombosis and Haemostasis*. 2010;8(11):2450-2457. doi:10.1111/j.1538-7836.2010.04044.x
4. Idraparinux versus Standard Therapy for Venous Thromboembolic Disease. *N Engl J Med*. 2007;357(11):1094-1104. doi:10.1056/NEJMoa064247
5. Méan M, Righini M, Jaeger K, et al. The Swiss cohort of elderly patients with venous thromboembolism (SWITCO65+): rationale and methodology. *J Thromb Thrombolysis*. 2013;36(4):475-483. doi:10.1007/s11239-013-0875-2
6. Tritschler T, Salvatore SP, Kahn SR, et al. ISTH definition of pulmonary embolism-related death and classification of the cause of death in venous thromboembolism studies: Validation in an autopsy cohort. *Journal of Thrombosis and Haemostasis*. 2021;19(10):2514-2521. doi:10.1111/jth.15458
7. Nendaz M, Spirk D, Kucher N, et al. Multicentre validation of the Geneva Risk Score for hospitalised medical patients at risk of venous thromboembolism: Explicit ASsessment of Thromboembolic RIsk and Prophylaxis for Medical PATients in SwitzErland (ESTIMATE). *Thromb Haemost*. 2014;111(03):531-538. doi:10.1160/TH13-05-0427
8. Spyropoulos AC, Anderson FA, FitzGerald G, et al. Predictive and Associative Models to Identify Hospitalized Medical Patients at Risk for VTE. *Chest*. 2011;140(3):706-714. doi:10.1378/chest.10-1944
9. Rosenberg D, Eichorn A, Alarcon M, McCullagh L, McGinn T, Spyropoulos AC. External Validation of the Risk Assessment Model of the International Medical Prevention Registry on Venous Thromboembolism (IMPROVE) for Medical Patients in a Tertiary Health System. *JAHA*. 2014;3(6):e001152. doi:10.1161/JAHA.114.001152
10. Schünemann HJ, Cushman M, Burnett AE, et al. American Society of Hematology 2018 guidelines for management of venous thromboembolism: prophylaxis for hospitalized and nonhospitalized medical patients. *Blood Advances*. 2018;2(22):3198-3225. doi:10.1182/bloodadvances.2018022954
11. Tapson VF, Decousus H, Pini M, et al. Venous Thromboembolism Prophylaxis in Acutely Ill Hospitalized Medical Patients. *Chest*. 2007;132(3):936-945. doi:10.1378/chest.06-2993

© 2024 Häfliger E et al. *JAMA Network Open.*
