# Supplementary material for: Risk Assessment Models for Venous Thromboembolism in Medical Inpatients
Source: JAMA Netw Open. 2024 May 10;7(5):e249980. doi: 10.1001/jamanetworkopen.2024.9980 (PMC11087835; doi:10.1001/jamanetworkopen.2024.9980)
Supplement: Supplement 2. — Data Sharing Statement [file jamanetwopen-e249980-s002.pdf]

Data Sharing Statement

Häfliger. Risk Assessment Models for Venous Thromboembolism in Medical Inpatients. *JAMA Netw Open*. Published May 10, 2024. doi:10.1001/jamanetworkopen.2024.9980

Data
Data available: Yes
Data types: Deidentified participant data, Data dictionary
How to access data: christine.baumgartner@insel.ch
When available: With publication

Supporting Documents
Document types: None

Additional Information
Who can access the data: researchers whose proposed use of the data has been approved
Types of analyses: for scientific purposes
Mechanisms of data availability: with a signed data access agreement
Any additional restrictions: approval by ethical committee required
